# Multi-Center Pre-clinical Consortia to Enhance Translation of Therapies and Biomarkers for Traumatic Brain Injury: Operation Brain Trauma Therapy and Beyond

**DOI:** 10.3389/fneur.2018.00640

**Published:** 2018-08-07

**Authors:** Patrick M. Kochanek, C. Edward Dixon, Stefania Mondello, Kevin K. K. Wang, Audrey Lafrenaye, Helen M. Bramlett, W. Dalton Dietrich, Ronald L. Hayes, Deborah A. Shear, Janice S. Gilsdorf, Michael Catania, Samuel M. Poloyac, Philip E. Empey, Travis C. Jackson, John T. Povlishock

**Affiliations:** ^1^Safar Center for Resuscitation Research, Department of Critical Care Medicine, University of Pittsburgh School of Medicine, Pittsburgh, PA, United States; ^2^Safar Center for Resuscitation Research, Department of Neurological Surgery, University of Pittsburgh, Pittsburgh, PA, United States; ^3^Department of Biomedical and Dental Sciences and Morphofunctional Imaging, University of Messina, Messina, Italy; ^4^Oasi Research Institute (IRCCS), Troina, Italy; ^5^Program for Neuroproteomics and Biomarkers Research, Departments of Psychiatry, Neuroscience, and Chemistry, University of Florida, Gainesville, FL, United States; ^6^Department of Anatomy and Neurobiology, Virginia Commonwealth University, Richmond, VA, United States; ^7^Department of Neurological Surgery, University of Miami Miller School of Medicine, Miami, FL, United States; ^8^Center for Innovative Research, Center for Neuroproteomics and Biomarkers Research, Banyan Biomarkers Research, Banyan Biomarkers, Inc., Alachua, FL, United States; ^9^Brain Trauma Neuroprotection and Neurorestoration Branch, Center for Military Psychiatry and Neuroscience, Walter Reed Army Institute of Research, Silver Spring, MD, United States; ^10^Banyan Biomarkers, Inc., San Diego, CA, United States; ^11^Department of Pharmacy and Therapeutics, Center for Clinical Pharmaceutical Sciences, University of Pittsburgh, Pittsburgh, PA, United States; ^12^Department of Pharmacy and Therapeutics, Center for Clinical Pharmaceutical Sciences and the Clinical Translational Science Institute, University of Pittsburgh, Pittsburgh, PA, United States

**Keywords:** biomarker, pre-clinical consortium, neuroprotection, drug screening, reproducibility, theranostic, rigor, target engagement

## Abstract

Current approaches have failed to yield success in the translation of neuroprotective therapies from the pre-clinical to the clinical arena for traumatic brain injury (TBI). Numerous explanations have been put forth in both the pre-clinical and clinical arenas. Operation Brain Trauma Therapy (OBTT), a pre-clinical therapy and biomarker screening consortium has, to date, evaluated 10 therapies and assessed three serum biomarkers in nearly 1,500 animals across three rat models and a micro pig model of TBI. OBTT provides a unique platform to exploit heterogeneity of TBI and execute the research needed to identify effective injury specific therapies toward precision medicine. It also represents one of the first multi-center pre-clinical consortia for TBI, and through its work has yielded insight into the challenges and opportunities of this approach. In this review, important concepts related to consortium infrastructure, modeling, therapy selection, dosing and target engagement, outcomes, analytical approaches, reproducibility, and standardization will be discussed, with a focus on strategies to embellish and improve the chances for future success. We also address issues spanning the continuum of care. Linking the findings of optimized pre-clinical consortia to novel clinical trial designs has great potential to help address the barriers in translation and produce successes in both therapy and biomarker development across the field of TBI and beyond.

## Introduction

New approaches are urgently needed to successfully translate therapies and biomarkers from the pre-clinical arena to therapeutic successes in clinical trials in the field of traumatic brain injury (TBI). For therapies, reviews have suggested countless explanations for these failures, usually placing the blame on issues related to clinical trial design, heterogeneity of patients, lack of accurate injury phenotyping, inadequate outcome assessment tools, and/or sub-optimal drug dosing, among other concerns (1–3). Clinical research in TBI has begun to take on the translational challenge and propose innovative approaches to address a number of the potential roadblocks to therapy development. For example, the emergence of several large multi-center clinical consortia in the field of TBI incorporating novel trial designs such as comparative effectiveness (4–6) and the development of phenotype-directed trials (7, 8), among others, are exciting developments. Challenges and limitations to pre-clinical study design could also underlie some of the failures in translation. Given many negative or inconclusive clinical trials and the well-recognized anatomical and pathogenetic heterogeneity of TBI phenotypes, it seemed logical to consider strategic alliances and collaborations capable of tackling these challenges through assembly of a multi-center pre-clinical consortium. To that end, a multi-center pre-clinical therapy and biomarker screening consortium, Operation Brain Trauma Therapy (OBTT) was developed, supported by the United States Department of Defense (DoD). Ten manuscripts, to date, have been published by OBTT including primary findings on individual therapies, reports on serum biomarkers, and reviews and overviews (9–18).

By using multiple pre-clinical models in a multi-center design, OBTT established two major goals for TBI therapy development and advancement, (1) to identify the most promising therapies–those with robust beneficial effects across models which might be successful across all TBI phenotypes in a conventional randomized controlled trial (RCT) in humans, and (2) to identify therapies that show model dependence which could help guide precision medicine based on therapeutic trials in patients with specific anatomical TBI phenotypes.

We also superimposed the assessment of serum biomarkers of brain injury, specifically two in current clinical development/testing (i.e., glial acidic fibrillary protein [GFAP] and ubiquitin carboxy-terminal hydrolase-1 [UCH-L1]), in an attempt to generate robust and rigorous pre-clinical evidence for their use as surrogate endpoints for predicting clinical outcomes and therapeutic benefit (i.e., testing their theranostic value). A second biomarker-related goal of OBTT is to create repositories of blood samples and brain tissue to facilitate opportunities for legacy research in order to test novel TBI biomarker candidates. OBTT's galvanizing efforts and accomplishments support the role and utility of pre-clinical consortia in TBI and suggests that OBTT has only scratched the surface of the potential of this approach. Several reviews and updates on the findings of OBTT have been published (10, 17, 18). In this review, we build on the lessons learned from the work of OBTT and focus on how its approach might be further harnessed to optimize future development of consortium-based therapy and biomarker screening to facilitate future translational successes.

## Considerations for designing the infrastructure of pre-clinical TBI consortia

### Screening models

Owing to the great heterogeneity of clinical TBI, a number of animal models mimicking the different aspects of human TBI have been developed. In OBTT, we chose to use three rodent TBI models, namely parasagittal fluid percussion injury (FPI), controlled cortical impact (CCI), and penetrating ballistic-like brain injury (PBBI), covering a spectrum of injury that included contusion, diffuse injury, and penetrating injury, respectively. These three models also represented the principle rat models being used in each of the three screening centers. Moreover, midline FPI in the micro pig was selected as the large animal model to test therapies, as it represented both a gyrencephalic TBI animal model and produced a mild diffuse injury, not captured in the rat models. Nevertheless, a number of additional modeling strategies could be adopted in an attempt to embellish OBTT, alter its scope, and/or craft a novel consortium targeting different facets of TBI and/or addressing different goals. For example, to more comprehensively address therapies across the full spectrum of severe TBI, consideration should be given to incorporating secondary insults such as hypoxemia, hemorrhage, and/or polytrauma, given their important contributions to unfavorable outcome (19, 20). Similarly, it might be of value to include one or more of the established mild TBI models into the OBTT design–or into one or more separate new TBI consortia. Such an approach might provide unique insight as to what secondary injury mechanisms may cross the spectrum of injury severity as a potential therapeutic target. As the DoD was instrumental at the inception of OBTT, the inclusion of a blast TBI model would represent another valuable addition to a new DoD funded TBI consortium. Another modeling strategy to consider would be to include more than one injury severity level in each model, an approach that is rarely taken even in individual laboratories. As injury severity can vary widely even within similar clinical TBI phenotypes, that approach might represent a valuable experimental framework to evaluate the performance of candidate therapies.

A focused pre-clinical consortium approach also lends itself to studying mild TBI and/or repetitive mild TBI. OBTT has directed all of its effort on assessing therapeutic effects on outcomes assessed over about 1 month after injury. Given the interest in long-term outcomes, chronic traumatic encephalopathy (CTE), and links between TBI and neurodegenerative diseases, studies assessing outcomes of much longer durations are needed. Studies to 1-year outcome have been carried out in both FPI and CCI (21, 22). Further discussion of the issue of outcome duration will follow in the section on therapeutic testing. Finally, OBTT, or other multi-center TBI consortia, could readily incorporate additional behavioral outcomes to study the link between TBI and posttraumatic stress disorder and its treatment.

Other aspects of modeling deserve consideration for incorporation into future multi-center TBI consortia. OBTT allowed each site to use their model as it was employed in current operation, without major modifications. This included approaches to anesthesia, analgesia, surgery, and most aspects of behavioral testing and histology. This approach was taken to ensure that the consortium was not bogged down in model development and could promptly launch therapy testing. Model development can produce challenges to successful transition of consortium development to therapeutic testing, as was seen in the pioneering work of the Multicenter Animal Spinal Cord Injury Studies (MASCIS) consortium (23). Among additional modeling issues that merit consideration are sex and age. In OBTT, young adult male rats were selected for the cross-model screening, given the preponderance of young adult males in TBI-related combat casualty care, germane to the goals of the DoD, which is funding the work. The choice of male rats was also influenced by the fact that most published studies testing therapies in pre-clinical models of TBI were carried out in young adult male rats, which was important for therapy selection and dosing in OBTT. However, differing approaches might be desirable depending on the target population. Moreover, research interpreting the effects of drugs in the context of sex and its impact on pathobiology represents another important step forward informing future clinical trial design.

### Efficacy endpoints in therapy screening

In OBTT, considerable thought was placed into designing an approach to compare therapeutic efficacy across models. For the rat studies, a 66 point scoring matrix was developed that weighted each model equally (a maximum of 22 points in each model), and addressed conventional outcomes already used at each site. Outcomes manageable in a screening approach were selected including assessments of motor testing, cognitive testing, and histology (10, 11).

- Motor testing: Since approaches to motor testing differed across centers, each site was allowed to use their established motor function tests.
- Cognitive testing: Morris water maze (MWM) testing was used to evaluate cognitive outcome at each site–given that it was already routinely applied to each rat model at each center. However, there were differences in the details of the approach to MWM testing at each site that were, once again, not altered. Given the importance of cognition to producing favorable outcomes in clinical trials, it was weighted the highest with a maximum of 10 of the 22 points in each model being allocated to MWM testing.
- Histology: Lesion volume and hemispheric (CCI and PBBI) or cortical (FPI) tissue loss were the histological outcomes scored for the rat studies in OBTT.

These outcome measures were selected given their extensive track record in the field of TBI and routine use at each site. Nevertheless, a host of additional cognitive and behavioral outcome assessment tools have been used in TBI and are available including fear conditioning, novel object recognition, open field testing, elevated plus maze, and forced swim testing among others (24). Given the extensive track record that the MWM has had in the field of TBI, and its routine use at each site, it was the logical choice. Nevertheless, a battery of cognitive outcome tasks might also provide a greater opportunity to detect beneficial effects given that robust beneficial effects of therapies on cognitive outcome have been surprisingly limited in the initial work of OBTT. A battery of cognitive outcome tests might also better reflect the functional recovery seen in humans–since the Glasgow Outcome Scale, the currently used outcome tool for clinical trials in severe TBI, is a general assessment tool–and its analog in rodent models remains undefined. Thus, beyond the screening approaches currently used by OBTT, alternative behavioral outcome tasks may be desirable, in future consortium designs.

Drug screening in our rat models might also benefit from the assessment of additional histological outcomes. Examples include assessment of hippocampal or cortical neuronal death–using either conventional histological approaches, or Fluoro-jade or NEU-N staining (20, 25), or assessing axonal injury using markers such as amyloid precursor protein, which are established outcomes in our micro pig model (26). Beyond simply using additional neuropathological readouts, more sophisticated approaches such as linking neuropathology to behavior, might be necessary to reveal beneficial effects for some therapies. For example, in work by Zhao et al. (27) assessing the efficacy of the cholinesterase inhibitor galantamine in CCI, preservation of GABAergic neurons in the dentate hilus was noted and specifically linked to improvements in hippocampally-mediated memory processing using contextual fear testing. Trade-offs resulting from the use of general screening vs. target-specific outcomes thus represent a challenge to designing a therapy screening consortium.

### Analytical approaches

In OBTT, conventional approaches to data analysis were taken using the same statistical software (SPSS) at each site. Standard statistical tests were applied to the data in each model, points for the effects (positive or negative) were tabulated in our outcome matrix (22 points for each model), and the results across models were summed to generate an overall score for each therapy (10, 11). This also allowed cross model comparisons of efficacy. We also used a pooled analysis of four pre-defined outcomes that were used at each site, namely, (1) average MWM latency, (2) percent time in target quadrant in the probe trial, (3) contusion volume and (4) total tissue loss in the injured hemisphere (CCI and PBBI) or cortex (FPI). This allowed for a direct comparison of models across four shared outcomes, providing a useful tool to monitor the stability of each model from study to study and show their reliability and reproducibility.

An additional innovative analytical approach that has not been taken thus far by OBTT, but has been successful in other studies, is a topological data analysis (TDA). This “big data” analytic approach was recently applied to an archived database from the MASCIS consortium (28). TDA of the MASCIS database was used to examine the impact of various factors associated with outcome in experimental spinal cord injury to reveal that peri-operative arterial hypertension was highly predictive of unfavorable outcome. It is thus appealing for applications in the design of future pre-clinical TBI consortia.

Finally, one of the surprising findings in OBTT has been the fact that so few of the drugs tested have shown robust efficacy, either across models or in individual models. Only two of the 10 drugs tested showed clear beneficial effects. Levetiracetam was beneficial in both FPI and CCI (17) while glibenclamide, in preliminary analysis, showed benefit specifically in CCI (18). Given these findings and the fact that our goal has been to search for therapies with the greatest likelihood of clinical success, an alternative analytical approach that may also be explored is to use a *P-*value of 0.1 rather than 0.05 as the threshold for defining an effect. The rationale for this approach centers on the fact that in a screening consortium the sample size is not statistically powered for each outcome, and with novel therapies there may not even be information on anticipated effect size. This may preclude definitive sample size calculations. Also, the optimal dose and route of administration for a given drug may differ across models. Comparing one or two doses of a drug using a single treatment protocol across models in screening thus limits the ability to optimize a given therapy in each model, potentially warranting application of a lower threshold for identifying therapeutic efficacy or priority. The trade-off to a lower statistical bar would be the potential for identifying therapies more likely to fail. Also, we do not believe that a large number of additional beneficial effects would emerge adding a *P*-value window from 0.05 to 0.1. Other innovative analytical approaches may also be informative mirroring the novel approaches to clinical trial design—such as adaptive trial design—that are now being used (29).

### Reproducibility in screening vs. a robust effect that defies the noise

Although much has been written about problems with clinical trial design as a cause for translational failure in TBI, concerns over reproducibility of pre-clinical studies may also play a role. This topic has been discussed in detail in the field of cancer research, where concerns over the inability to reproduce numerous pre-clinical reports in high impact journals spawned the term “reproducibility crisis” (30, 31). The purpose of OBTT was not to serve as a tool to evaluate reproducibility of published pre-clinical investigations; however, given that the published pre-clinical work importantly guided therapy selection and dosing, and that efforts toward achieving a high level of rigor were substantial, its findings by default provide some insight into the issue of reproducibility. Use of common data elements can help to maximize the chance of reproducing published findings (24), however, extremely subtle methodological differences between protocols can greatly affect findings (32). In OBTT even if the model and dosing selected was identical to that used in published reports, many other discrepant factors may have influenced the findings including issues such as differences in anesthesia, animal strain, vendor, age, diet, surgical approach, brain temperature, details of the injury, and others. Lithgow et al. (32) in a recent commentary stated that it is a rare project that specifies methods with a high level of precision and that standardization may be counterproductive–suggesting that it may be better to focus on highly robust results that persist across a wide range of conditions than to chase fragile findings that occur only within narrow parameters. Such an approach mirrors that taken by OBTT, where rather than testing reproducibility, therapeutic efficacy across multiple established models is sought. Given that both anatomical TBI phenotypes and injury severity vary greatly within clinical trials, such an approach seems justified for therapy screening.

### Monitoring consortium stability and performance

Appropriately designed multi-center pre-clinical consortia allow for rigorous protocolized comparisons of therapies and biomarkers across multiple models and also provide unique insight into the pathophysiology of TBI through direct model comparisons. Beyond simply testing of therapies and biomarkers and comparing models, the consortium approach also allows monitoring of model stability and performance. To optimize comparison of multiple therapies tested in multiple models, it is essential that model stability be monitored, given that in a consortium like OBTT, years are required to carry out demanding therapeutic *in vivo* studies. Model stability, defined as a given model's ability to produce the same magnitude of injury response over time, can be influenced by staff changes, mechanical wear on the injury device, and other factors in the laboratory environment such as alterations that impact the microbiome, temperature, lighting, or other factors. Pooled analysis of four key TBI outcomes across models for each therapy tested by OBTT not only allows for an objective comparison of the therapies, but also allows for an assessment of temporal stability of each model by comparing outcomes in the TBI vehicle group for each model in each study. This ensures that a stable and appropriate therapeutic target is generated and allows minor discrepancies to be addressed if slight changes in model severity are seen over time.

### The unspoken challenge: transparent reporting and publishing negative results

OBTT represents a new rigorous paradigm-changing approach to identify neuroprotective therapies for clinical TBI. As such, the bar for performing the studies, reporting the data and presenting the results has been appropriately raised. The OBTT investigators are committed to standards such as use of a manual of standard operating procedure and publication of all findings regardless of the outcome (11–14). It may be noteworthy that the disappointing results seen by OBTT across models for treatment with erythropoietin (EPO) were also seen in subsequent clinical trials (33, 34). However, our goal is not to be prescriptive or proscriptive, recognizing the many limitations inherent in therapy screening strategies, especially across models. Our goal is simply to carry out high quality, rigorous, and timely screening studies of therapeutic efficacy across multiple models to advance therapies to successful clinical trials either across TBI phenotypes, or in a precision-based clinical trial.

### Considerations for advancement to a gyrencephalic TBI model

In OBTT, therapies and biomarkers showing promise are advanced to testing in a large animal, gyrencephalic, pre-clinical TBI model, namely midline FPI in micro pigs. Taking an approach that includes a second tier of therapy screening in a higher order animal model is logical, given the likelihood of gaining additional translational insight into a given therapy by carrying out studies in multiple species, including one with a gyrencephalic brain. This approach also addresses the practical issue regarding the high cost of carrying out initial therapy screening in large animal models. An approach such as the one taken by OBTT, has been outlined and updated in the RIGOR guidelines for stroke (35). Using a gyrencephalic animal may be of even greater importance in TBI than stroke given the key role of traumatic axonal injury in contributing to pathological progression and subsequent outcomes after TBI (36). The fact that in TBI neuropathology in long-term sequelae such as CTE, is prominent within the sulci, where mechanical strain and strain rate are hypothesized to be greatest in the gyrencephalic human brain, also reflects the usefulness of assessing gyrencephalic animal models of TBI prior to clinical translation (37). A large animal model can also facilitate the use of physiological monitoring, such as assessment of intracranial pressure or partial pressure of brain tissue oxygen (mirroring clinical care), and more extensive blood sampling. Large animal models may, in some cases, also require dosing paradigms that may more closely reflect the human condition and the immune system in rodents differs importantly from human (38, 39). Although other gyrencephalic species have been used on a sporadic basis, potentially due to the neuroanatomical and immunological similarities to humans, studies in pigs or micro pigs, have been used in the majority of large animal TBI investigations (40). Use of computational modeling of the key factors effecting drug response (i.e., allometric scaling) across more than one species is the more accurate method for estimating human equivalent dosing. Although there is added expense in the assessment of multiple species, such methods improve the accuracy of estimation of key factors of drug disposition (41, 42). Recent, preliminary studies have suggested that the ferret may represent a lower-order gyrencephalic species that deserves consideration (43). Indeed, the original development of the CCI model was carried out in ferrets (44). However, work to date in ferrets has been limited with regard to two key facets of therapy screening. First, behavioral outcome characterization in ferrets has been exploratory in nature, even in studies using the CCI model (43). Second, there is little work evaluating potential therapies in ferret TBI models, therefore, substituting our pre-clinical rat studies with ferrets would be impractical. Also, as there is limited support to either substantiate or refute potential therapeutic efficacy in either humans or large animal models following the detection of benefit in rodent preclinical models, the approach taken by OBTT of carrying out initial screening in rat models then advancing the therapies with promising findings in those rat models to pigs or other large animals is logical. However, a recent publication in *Nature*, suggests that for drugs targeting hippocampal neurogenesis, studies in rodents may be misleading, since the mechanism affected in rodents is not present in the human hippocampus (45). Finally, parallel studies in rodent and large animal models might represent a reasonable alternative screening strategy (46), however that approach does not address the prohibitive cost associated with the substantial numbers of large animals required for large animal screening.

## How do we select the best possible therapies to advance from the bench to the bedside?

In clinical trials across the field of acute brain injury including TBI, stroke, and global ischemia from cardiac arrest, and other conditions, there has been a consistent lack of successful RCTs testing novel pharmacological agents. Issues such as heterogeneity of the insult mechanism and severity, age, gender, and insensitive outcome assessment tools in humans have been implicated as reasons for these failures. However, recent highly successful studies in stroke assessing the efficacy of clot retrieval provide insight into considerations for the design and goals of pre-clinical consortia across the field of acute brain injury. Multiple RCTs of clot retrieval have reported highly significant benefit in stroke, with huge effect sizes >30% (47, 48). Indeed, some of the trials have been stopped early because therapeutic efficacy was shown with fewer patients than anticipated (47, 48). This suggests that a key to overcome the inherent “noise” in studies of TBI is to have a therapy with a large effect size. It is unclear whether or not any pharmacological approach in TBI can produce an effect size that matches the impact of rapid reperfusion (resulting from clot retrieval) vs. no reperfusion in stroke. However, it suggests that in TBI, a rigorous consortium-based approach using multiple models to identify highly robust therapies may be essential to achieving that goal. There are many aspects of therapy selection that merit discussion and careful consideration for a consortium approach moving forward.

### Literature based vs. high throughput screening based therapy selection

**Figure 1:**
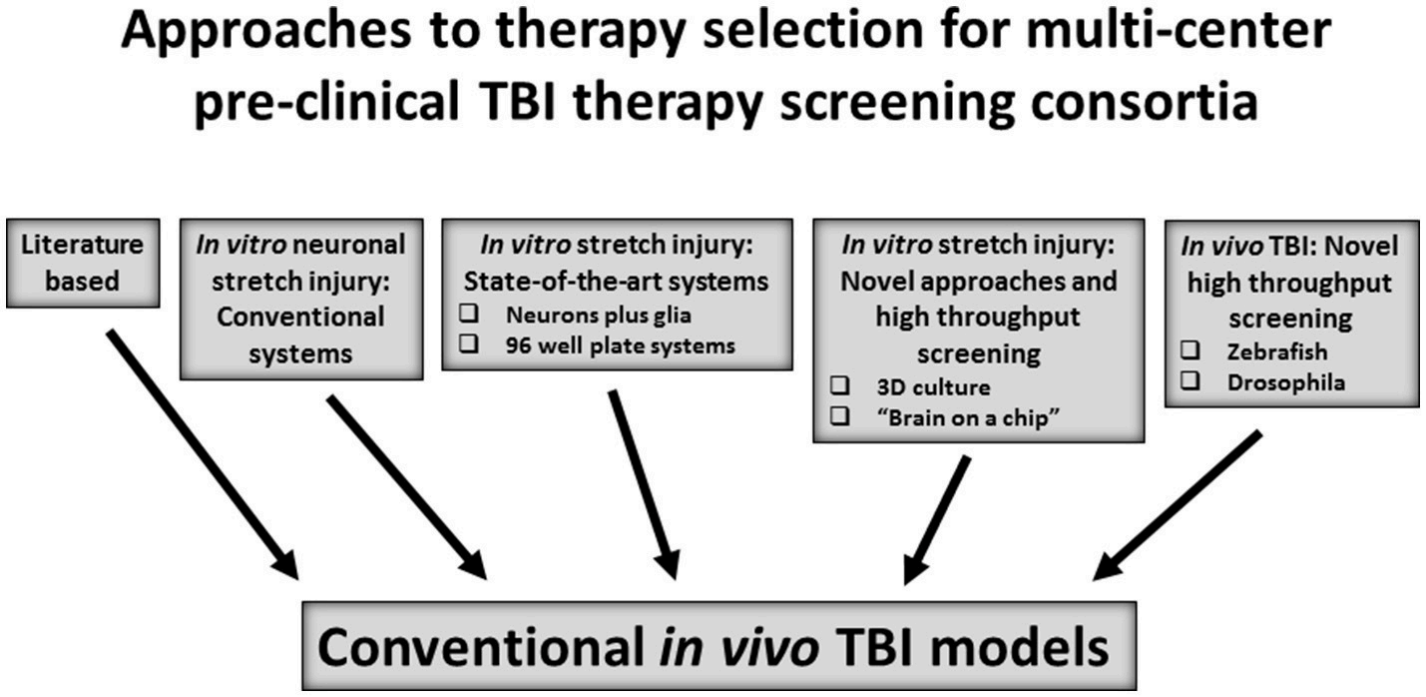
Approaches to select and/or advance therapies to *in vivo* therapeutic screening in conventional rodent and/or large animal models of traumatic brain injury (TBI).

In OBTT, a literature-based approach was used for therapy selection for testing in screening across the rat models. After a comprehensive literature review and consideration of recommendations from the OBTT investigators, advisory board, and programs in the DoD, a table of potential therapies with a description of the published studies in pre-clinical models of TBI was provided to the site principal investigators in OBTT and a secret ballot vote was taken to rank the therapies. This was followed by a discussion and final ranking of those therapies each year at an OBTT investigators meeting that was held at the annual meeting of the National Neurotrauma Society. Generally 3-4 therapies were selected for testing each year. This approach allowed the consortium to leverage the published literature, which for many of the therapies was fairly extensive. Nevertheless, it is not fully systematic and is challenged by the many differences between published studies in dosing and treatment protocols, species, outcomes, and other parameters–making it difficult to rank therapies objectively in either a quantitative or qualitative manner. An alternative strategy, or one that may be able to be coupled to a literature based approach is to consider the use of drug screening first in an established *in vitro* screening TBI model, such as stretch injury in neuron or neuron/glial cultures (49–51). In addition to standard approaches targeting neuronal death, novel *in vitro* approaches, to more closely mimic the *in vivo* environment in neuron/glial stretch models have suggested exciting profiles that can highlight axonal injury without appreciable neuronal death (52). More sophisticated systems biology models, such as 3D cell culture and “organ on a chip” approaches are used in cancer biology and liver disease to screen therapies (53–55). A high throughput screen was reported using induced pluripotent stem cells as a source of neurons in a model system to screen therapies against tauopathies in Alzheimer's disease (56). Similarly, neuronal stretch in a 96 well plate format has also been reported (57). Obviously, *in vitro* screening approaches are limited in their ability to incorporate clinically relevant features of TBI *in vivo* such as alterations in perfusion, ICP, inflammation, and other extra-cerebral factors, such as the microbiome, but they have the potential to screen and compare thousands of agents, including both those with strong literature support along with highly novel therapies. More advanced high throughput *in vivo* screening for leukemia has been carried out in zebrafish and, although exploratory as a tool for TBI, several reports of TBI modeling in zebrafish have been published (58, 59). Similarly, invertebrate TBI models such as in drosophila could be considered (60). The concept of incorporating an *in vitro* or other higher throughput screening strategy merits consideration. A paradigm illustrating options for therapy selection is shown in Figure 1.

## New horizons for testing therapies and biomarkers by pre-clinical consortia: defining the right therapeutic targets and monitoring target engagement

### A multi-model consortium-based screening approach may be essential to successful therapy development for a traditional RCT

In selecting therapies for screening by OBTT, a powerful influence has been the pressing need for new neuroprotective agents that can be rapidly translated to clinical trials. Therapies with pre-clinical literature support in one or more established models and that represented “low hanging fruit” i.e., drugs that are already FDA approved for other uses, were considered prime candidates. If successful, they could be rapidly brought to clinical trials, given that general drug safety was established. Often these drugs have effects (many of which represent “off target” effects of the drug's originally intended use) that produce neuroprotection in pre-clinical studies and might translate to benefit in human TBI. Many of these therapies have pleiotropic beneficial effects, such as targeting inflammation, mitochondrial failure, neuronal death, oxidative stress, or regeneration. Given the multifaceted secondary injury response to TBI, drugs with many potential therapeutic targets are alluring. However, they can present critical challenges for therapy development–in both the multi-model consortium setting and in clinical trials. For therapies that target multiple mechanisms, it may be unclear what mechanisms are critical to their neuroprotective effects. EPO and progesterone are examples of agents with pluripotent effects that have produced success in pre-clinical studies but have not translated successfully to humans (33, 34, 61). Neuroprotection by a given therapy may also be mediated by a different spectrum of effects in different models. The amount of blood-brain barrier (BBB) permeability, which can affect drug penetration into the brain, also differs across TBI models and varies as a function of injury severity, brain region, and time after injury even within a given model. A similar case exists for cerebral perfusion, which can be compromised to different degrees after injury in different models and in different brain regions in the same model (62–64). Thus, the amount and distribution of a given therapy in the injured brain after systemic administration may differ in each model. Layered upon this, the mechanism (or mechanisms) being targeted may vary in importance across models, and across brain regions and injury severity levels even in a given model. This can create major challenges for primary screening of therapies across TBI models, unless the drug being tested is one that has a high degree of BBB permeability, is highly potent, and/or has low toxicity–such that the necessary brain exposure can be achieved. It may be more than coincidence that the two drugs tested by OBTT with the greatest efficacy, levetiracetam and glibenclamide both have excellent BBB penetration and limited systemic toxicity. Although the issues of model and injury severity impacting both the mechanistic targets and drug delivery to brain might seem to represent a limitation for the pre-clinical consortium approach, these same challenges are seen in traditional RCTs in severe TBI, which feature heterogeneity of anatomical and pathological phenotypes, injury mechanism, injury severity, BBB permeability, perfusion, edema, and axonal injury, among other factors. Thus, if the goal is that of therapy development for a traditional clinical trial in severe TBI, for example enrolling patients with a Glasgow coma scale (GCS) score of 3 to 8, the multi-model pre-clinical consortium approach would seem to be appropriate. What approaches might best address treatment protocol design given these challenges?

In primary screening strategies, mechanism-based studies are generally not the goal, rather, clues as to potential efficacy, either in a single model or multiple models are sought to prompt more complete exploration of promising candidates. In OBTT a single literature based protocol, usually testing two doses and a vehicle group and sham, was evaluated across rat models. That approach allowed for therapies to be compared when administered in an identical manner across models. Although this allowed for rigorous comparisons, it is clear from the discussion above, that the dose and treatment regimen might be optimal for one model but suboptimal for another, depending on the studies in the literature on which dosing was based. In contrast, one might argue that, if a wide dose range was studied, and the therapy was reasonably non-toxic even at high doses, potential efficacy for use in a conventional RCT in TBI might be well defined. The strategy that we implemented, however, may have its greatest potential to identify therapies with efficacy in specific TBI phenotypes. The biggest challenge to therapy screening in multiple models is selecting the doses and treatment protocols. Many other strategies to dose and treatment protocol selection could be used for multi-model consortium based therapy screening in TBI. For example, in studies targeting the pre-clinical development of therapies for pediatric TBI, Kilbaugh et al. (44) used rodent and piglet models concurrently focusing on a single therapy (cyclosporin A), studying a range of doses, and selecting effects on mitochondria as the target mechanism. Cyclosporin A, in OBTT showed considerable model dependence in effects, ranging from modest benefit in the FPI model, no benefit in CCI, and toxicity in PBBI (13)–suggesting that dose, timing, and duration of treatment could be very challenging to optimize in human TBI, unless a specific TBI phenotype and injury severity level were targeted (65). Cyclosporin A indeed has considerable toxicity with use in humans (66). Rather than using a literature-based approach for drug dosing, studies could be carried out to generate serum, plasma, or CSF exposures to better target one or more putative mechanisms. However, a thorough assessment of pharmacokinetics and pharmacodynamics, including studies in brain, are generally beyond the scope of primary therapy screening by a consortium. Indeed, assessment of drug or brain tissue levels was surprisingly rare even in the studies in the literature upon which the treatment protocols were based in OBTT. Nevertheless, recognizing the failure to detect robust benefit for 4 of the initial 5 drugs tested, OBTT chose to directly address this for therapies 6-10. We measured serum drug levels in separate cohorts of treated rats in 3 of those therapies (i.e., glibenclamide, AER-271, and minocycline) to optimize the treatment protocols. Levels were not measured for amantadine, owing to the substantial pre-clinical literature base for its testing in rats and for VA64—given that it is a polymer. The best approach to dosing and protocol selection for drug testing in TBI by a pre-clinical consortium merits great consideration. Additional considerations are discussed below.

### Serum biomarker assessments of efficacy and target engagement

OBTT has provided considerable insight into an additional strategy to monitor and optimize dosing and treatment protocols, and to promptly evaluate efficacy in screening via the use of target engagement biomarkers. In screening, although definitive mechanism-based studies are not the goal, a rapid assessment of either overall potential efficacy or some evidence that the mechanistic target for a given therapy is being modulated is desirable. Throughout the screening carried out in rat models, serum biomarker levels were serially assessed with the goals of (1) comparing the biomarker profile across models and (2) exploring the theranostic potential of biomarkers in therapy screening (16). The results of OBTT's primary screening in rat models support the use of the serum biomarkers GFAP and UCH-L1 as TBI diagnostics. They were useful across models and assessments at 4 or 24 h after injury corroborated injury severity, correlated with cognitive deficits assessed between 13 and 21 days after injury, and predicted ultimate lesion volume and brain tissue loss, assessed at 21 days after injury (16). The associations were strongest in CCI. The data generated by OBTT were submitted and reviewed by the FDA and viewed as an important preclinical component of the total submission package for clinical development. GFAP and UCH-L1 were recently approved for clinical use. More important to the development of novel theranostics for rapid *in vivo* drug screening in TBI, GFAP showed promise in predicting therapeutic efficacy, notably predicting contusion volume and/or tissue loss (14–16). For example, levetiracetam's effect on hemispheric tissue loss at 21 days after CCI was predicted by 24 h serum GFAP levels. Thus, serum biomarkers have the potential to serve as early post injury indicators of therapeutic efficacy. This approach is already being used by others (67). GFAP has also been shown to be valuable in identifying and monitoring adverse effects associated with drugs tested by OBTT (13, 14). Thus, GFAP has the potential to address the need for sensitive preclinical safety biomarkers and be implemented in clinical trials and regulatory pathways for therapy testing. Given the efforts by the DoD to develop serum GFAP and UCH-L1 for use in combat casualty care at the time OBTT was launched, it was logical to begin with those two biomarkers in the work of OBTT. Other markers such as neuron specific enolase and S100β, along with novel markers (discussed later) merit study within the pre-clinical consortium framework. Serum biomarkers could serve in an additional capacity, germane to therapy screening in individual models in multi-model consortia, namely, as readouts of successful target engagement. For example, OBTT recently reported that serum levels of phospho-neurofilament-H (pNF-H), a marker of axonal injury, were also reduced by treatment with levetiracetam (68). This suggests that pNF-H may represent an example of a target-engagement biomarker to rapidly assess therapies specifically targeting axonal injury and/or contribute to understanding of how therapies primarily targeting other mechanisms impact axonal injury. Additional serum target engagement biomarkers could also prove useful as early readouts for therapy screening. For example, the cardiolipin lipid profile of brain mitochondria is unique, and serum levels of brain specific cardiolipins at 24 h after TBI could be used to screen therapies targeting brain mitochondrial injury (69). Other target engagement serum biomarkers merit exploration for TBI therapy screening such as those monitoring inflammation, BBB disruption, or synaptic injury, among others. Beyond using serum, it is also possible that magnetic resonance imaging could be used to screen for target engagement efficacy, such as for drugs targeting inflammation (70), although issues of cost and throughput could be challenging. In any case, evidence of mechanistic efficacy to complement conventional outcomes could greatly enhance therapy screening in a multi-model pre-clinical approach.

## New horizons for testing therapies and biomarkers by pre-clinical consortia: phenotype based therapies

Treatments for TBI may need to be phenotype specific. This concept has been discussed frequently for severe TBI, where experts in the field often use the example of multiple patients with highly different pathologies on admission computed tomographic scans are all being administered the same therapy in RCTs (71). Phenotype based multi-center therapy screening may need to be linked to phenotype based clinical trials. One could envisage that this could be efficient on multiple fronts, including (1) directing therapy selection for screening based on the specific pathophysiologic mechanisms of the TBI phenotype, (2) guiding dosing and treatment protocol selection based on the time course of the key secondary injury mechanisms in that phenotype and the required drug exposure to alter that mechanism, and (3) selecting the most clinically relevant outcomes in the pre-clinical models based on the phenotype. For example, a new therapy to reduce ICP might be able to be efficiently developed in a consortium by targeting brain edema that develops in a specific TBI phenotype such as contusion. That approach would still not resolve the contribution of genetics, epigenetics, or extra-cerebral confounders (72, 73), but could address many challenging issues in consortium based screening and clinical RCTs. Phenotype-based therapies are particularly important to mild TBI, where divergent behavioral sequelae such as cognitive dysfunction, PTSD, sleep disorders, headache, and depression, among others are the therapeutic targets (74). Thus a phenotype-based screening approach is likely essential in mild and mild repetitive TBI. This approach could also be informative to serum biomarker development in TBI, since pre-clinical models with specific phenotypes might be able to help unravel the contribution of various insults in patients with complex pathologies.

## New horizons for testing therapies by pre-clinical consortia: are drugs the answer?

With the exception of levetiracetam and glibenclamide, the limited efficacy of the initial therapies tested by OBTT (nicotinamide, EPO, cyclosporin A, simvastatin, kollidon VA64, amantadine, minocycline, and E64d) has been surprising. Given the demands that showing efficacy across multiple models placed on a rigorous screening approach, this may not be surprising. But given the failure of multiple RCTs of drugs such as EPO (33, 34, 61), it may be that our approach is optimal for developing therapies to be tested in conventional RCTs of acute therapies in severe TBI. However, beyond the approach discussed above for phenotype based drug screening, it may be that for rigorous multi-model therapy testing, alternatives to drugs are needed. Combination therapy may also be necessary. However, since clear efficacy of individual agents has been difficult to confirm, the selection of drug combinations is challenging. Strategies such as combining a therapy showing efficacy on behavior with one that improves histology could be optimal. Combining drugs that target divergent or similar mechanisms, seeking additive or synergistic effects, might also be optimal. However, the approach to dosing in combination therapy requires considerable expertise (75). Many trials of combination therapy in pre-clinical models of TBI have failed or shown that benefit of one agent is negated by a combination approach (76). Given the need for a robust therapy, that penetrates the brain, and has limited toxicity, it may be that approaches beyond drug therapy are needed, such as cellular therapies (77), nanoparticles (78), or manipulation of the microbiome (79). A detailed discussion of innovative therapies for TBI, however, is beyond the scope of this review (36, 80, 81).

## New horizons for testing therapies and biomarkers by pre-clinical consortia: beyond acute therapies

**Figure 2:**
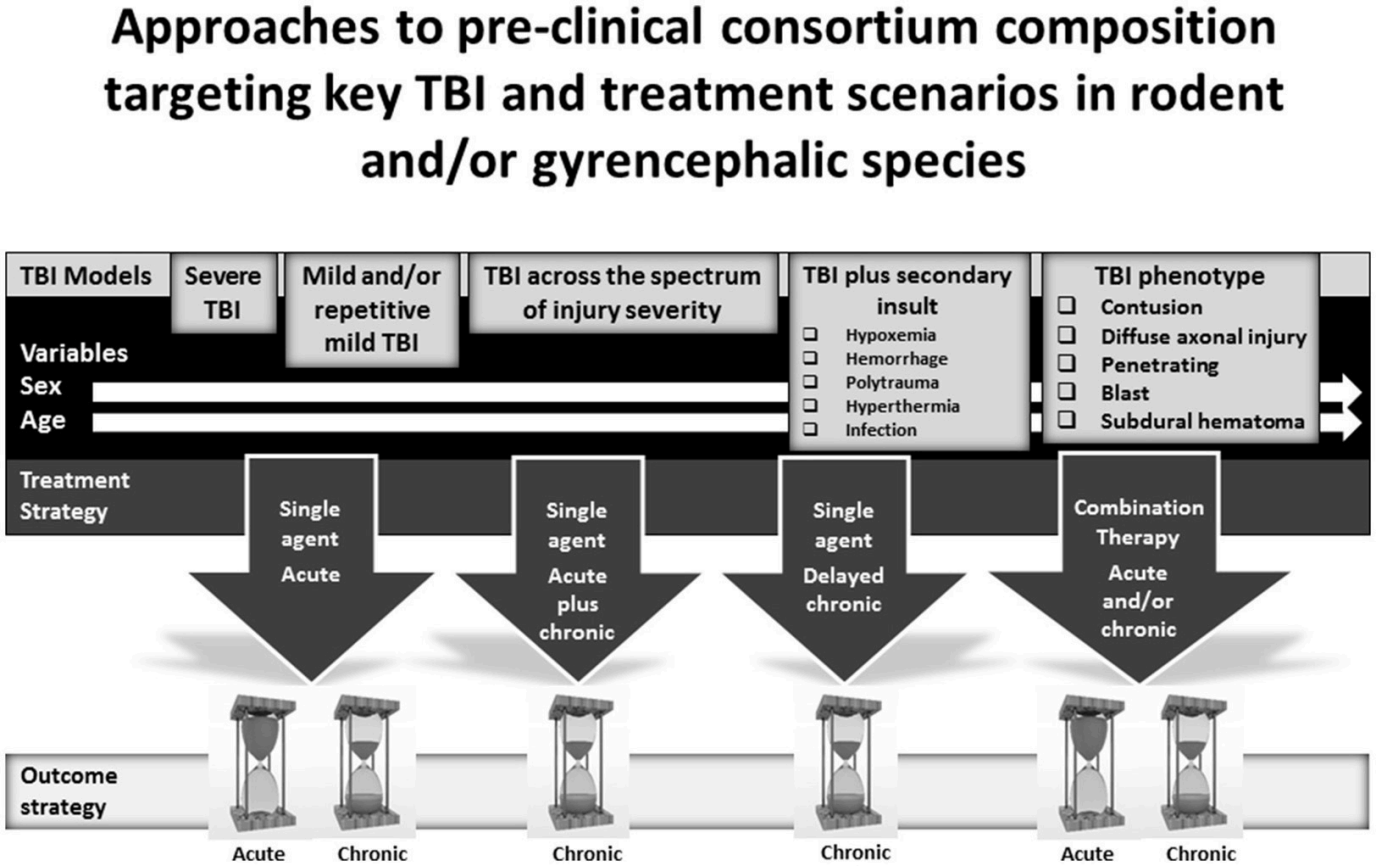
Potential compositions of pre-clinical consortia for screening therapies for the treatment of traumatic brain injury (TBI) including (1) models targeting mild or severe TBI, secondary insults, and TBI phenotypes, (2) treatment strategies, and (3) evaluation of early or long-term outcomes.

OBTT has focused on the development of acute therapies for severe TBI. However, there are other exciting possibilities for pre-clinical therapy screening using a multi-center, multi-model or phenotype based approach. The most obvious potential opportunity for pre-clinical consortium development is in the study of mild and/or mild repetitive TBI. A host of mild TBI models are available and several therapies have shown benefit in these models (82–85). Similarly, given the importance of long-term outcomes and the link and common mechanisms underlying TBI and neurodegenerative diseases, it would be exciting to create long-term outcome oriented consortia capable of testing therapies to mitigate or prevent neurodegeneration. Seminal reports in the CCI and FPI models on 1-year chronic outcomes revealed dramatic targets including progressive tissue loss and persistent cognitive deficits (21, 22), setting the stage for similar studies in mild and repetitive mild TBI models. The approach to therapy testing in this setting could include (1) acute treatment, (2) delayed chronic treatment, and (3) acute plus chronic therapy. Some work on TBI in individual centers has begun to use these types of approaches (86). Long term studies would represent perfect opportunities to evaluate the impact of enriched environment with and without pharmacotherapy, mimicking clinical TBI rehabilitation (87). Conventional outcomes and those germane to chronic neurodegeneration, plasticity, and chronic neuro-inflammation, should be included. Consortia addressing long-term outcome therapy testing could also guide biomarker development, given the need for biomarkers linking acute injury with chronic TBI pathologies and neurodegenerative disease, an area that is only beginning to be explored (88). Potential approaches to pre-clinical consortium composition targeting key TBI and treatment scenarios in rodent and/or gyrencephalic species are shown in Figure 2.

## New horizons for therapy and biomarker development by pre-clinical consortia: partnering with pharma

Despite legal, ethical, and financial implications, public-private partnerships that allow the pooling of expertise, resources and funding, as well as providing cross-fertilization, are gaining momentum and strongly encouraged by government agencies including NIH (89). Collaborative projects involving industry and academics represent a unique conceptual framework and a promising cost-effective opportunity–risk and reward sharing approach. They may represent a logical avenue to consider in future therapy testing. For example, given the putative key role of cerebral edema in secondary injury after severe TBI, OBTT tested the aquaporin-4 antagonist AER-271 (Aeromics Inc.), a novel proprietary drug, in primary screening in rats (as therapy number 8 tested), navigating the necessary legal and administrative issues required for such a multi-center, DoD-funded partnership with the pharma (18).

## Conclusions

### Linking the findings of pre-clinical consortia to optimized clinical trial design

**Figure 3:**
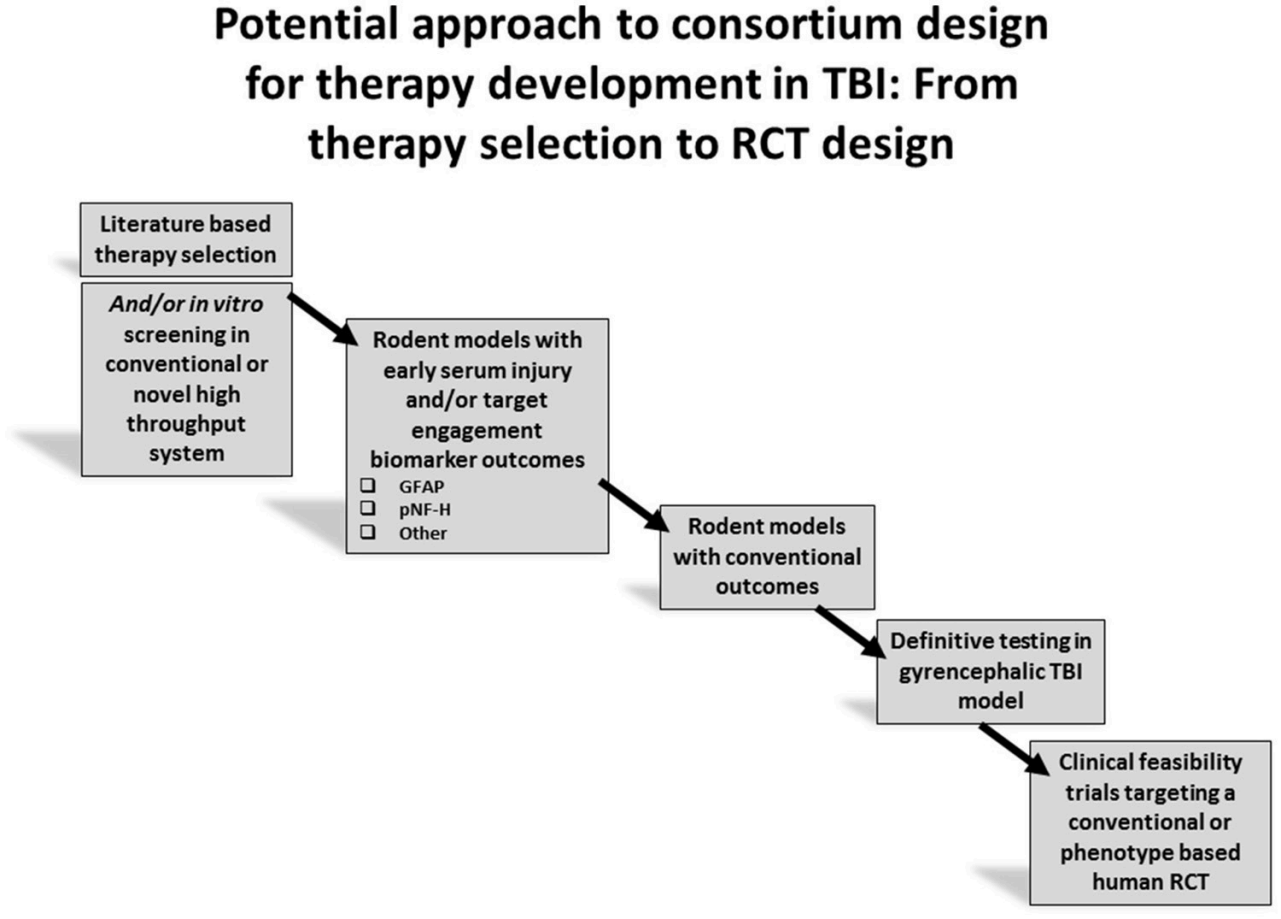
Overall strategy for therapy development using target engagement biomarkers to aid in screening prior to advancing to definitive studies of long term outcome.

As pre-clinical drug development is evolving, and the novel strategies proposed in this review are advanced, clinical trials are also experiencing a number of advances in design. In severe TBI, comparative effectiveness approaches are being carried out in large numbers of patients in both adults and children (4–6). Novel clinical trial design, such as adaptive designs, where computer-driven randomization algorithms allow for the study of multiple therapies simultaneously and with greatly reduced sample sizes (29, 90) and large clinical studies based on big data approaches, are gaining utility in TBI (91, 92). Finally, in mild TBI, exciting new phenotype-based approaches are underway, including approaches such as TRACK TBI and TEAM TBI (74, 93). The intersection between novel pre-clinical consortia and emerging advanced clinical investigations has potential for breakthroughs in TBI therapy across the spectrum of injury severity. A synopsis paradigm outlining an overall potential approach to consortium design for therapy development in TBI is provided in Figure 3.

## Author contributions

PK wrote the original draft, assembled and incorporated comments from the co-authors and crafted the final draft. All of the other co-authors contributed to manuscript review and revision.

### Conflict of interest statement

RH owns stock and is an officer of Banyan Biomarkers Inc. RH is an employee and receives salary and stock options from Banyan Biomarkers Inc. KW is a former employee of Banyan Biomarkers Inc. and owns stock. RH and KW also receive royalties from licensing fees and as such they may benefit financially as a result of the outcomes of the research reported in this publication. MC is an employee of and receives salary and stock options from Banyan Biomarkers Inc. The remaining authors declare that the research was conducted in the absence of any commercial or financial relationships that could be construed as a potential conflict of interest.
